# Isoamyl isothiocyanate preserves postharvest quality of matsutake (*Tricholoma matsutake*) by modulating oxidative and antioxidative homeostasis

**DOI:** 10.3389/fpls.2025.1627772

**Published:** 2025-07-22

**Authors:** Ruijie Dong, Wenhuai Tian, Essam ElShamey, Lei Xiao, Yue Xiao, Fadan Li, Zhifeng Chen, Dongmei Ye, Huayi Wang, Tong Zhu, Yumei Ding

**Affiliations:** ^1^ College of Food Science and Technology, Yunnan Agricultural University, Kunming, China; ^2^ Field Crops Research Institute, Agricultural Research Center, Cairo, Egypt; ^3^ College of Horticulture and Landscape, Yunnan Agricultural University, Kunming, China

**Keywords:** *Tricholoma matsutake*, isoamyl isothiocyanate, antioxidants enzymes, antioxidative homeostasis, postharvest quality

## Abstract

Water loss, browning, and softening are significant contributors to postharvest economic losses in matsutake. To effectively and safely preserve the quality of matsutake, the optimal concentration of isoamyl isothiocyanate (IAITC) was determined. Matsutake that had just been harvested were put in plastic containers and exposed to IAITC for 15 minutes at 25 ± 2°C, at different concentrations of (0, 10, 30, and 60 μLL^-1^). And then, the matsutake were stored for 8 days at 5.6 ± 0.6°C and 75 ± 5% relative humidity. Over the course of the storage period, the following parameters were tracked, including changes in firmness, weight loss rate, chitin content, the activities of superoxide dismutase (SOD), catalase (CAT), peroxidase (POD), polyphenol oxidase (PPO), total phenolics and ascorbic acid (ASA). These findings showed that IAITC therapy at a dose of 10 μLL^-1^ considerably decreased weight loss, increased the activity of antioxidant enzymes, and maintained antioxidant content. Additionally, this treatment inhibited PPO activity, decreased malondialdehyde (MDA) accumulation, and maintained chitin content, thereby ensuring the integrity of the cell membranes. These changes contributed to the preservation of both the sensory and nutritional quality of the matsutake. In conclusion, IAITC treatment primarily enhanced antioxidant activity and reduced oxidative damage, thereby delaying the senescence of matsutake and maintaining their quality. This study provides novel insights into the biological mechanisms underlying postharvest preservation using IAITC and lays the foundation for further research and the promotion of its application in matsutake mushroom postharvest management.

## 1 Introduction

Matsutake (*Tricholoma matsutake*) S. Ito & S. Imai Singer, one of the rarest and most valuable natural medicinal mushrooms in the world, often hails as the “king of mushrooms”. It is rich in vitamins, proteins, polysaccharides, amino acid and other bioactive compounds (Wang, 2020). It exhibits various health benefits, including enhancing physical strength, boosting the immune system, possessing antioxidative properties, and exhibiting anti-tumor effects (Kim et al., 2009). Moreover, it is a rare and highly sought-after wild edible fungus that cannot be artificially cultivated.

Matsutake mushrooms possess a high in water content, and throughout the distribution process, they continue to exhibit significant physiological and respiratory activities. These processes lead to the degradation of their nutritional components, resulting in aging, browning, flavor changes, waterlogging of tissues, and microbial growth on their surface (Wang, 2013; Yang, 2015). Besides, the decay of matsutake fruitbody caused by microbiota invasion was the major postharvest the quality deterioration. Preservation methods for matsutake primarily focus on postharvest physiological preservation. Currently, widely utilized preservation techniques for edible fungi include chemical, physical, and biological preservation methods (He et al., 2024). For example, it found that ultraviolet light combined with a 3% hydrogen peroxide solution for cleaning edible fungi effectively inhibited mold growth (Guan et al., 2013). Low-temperature (4°C) treatments have been shown to delay aging, browning, and reduce metabolic activities while slowing microbial growth in black boletus (Kong et al., 2025). It reported that pulsed light treatment significantly reduced water loss during storage and delayed browning in Pleurotus ostreatus during the later stages of storage (Wang et al., 2025). Furthermore, a composite coating film consisting of slime peptide and sodium alginate helped preserve the shiitake mushrooms’ quality and prolong their shelf life (Qian et al., 2024). The treatment of *Agaricus bisporus* with equal amount 1:1 compounds of essential oil and sodium alginate to form a film significantly reduced the respiration rate, weight loss, and polyphenol oxidase (PPO) activity of the mushrooms compared to other treatments, this approach increased mushroom firmness, color, total phenolics content, and antioxidant capacity, thereby significantly improving their quality (Eliezer et al., 2021).

The general formula for isothiocyanates (ITCs) is R-N=C=S, are isomers of thiocyanates belonging to heteronuclear cumulative dienes which are a class of sulfur and nitrogen-containing compounds. Natural ITCs were primarily discovered from cruciferous plants which were produced as secondary metabolites when encountering biological stress, ITCs were generated through direct interaction with enzyme of sinigrinase within cells, which catalyzes the hydrolysis of glucosinolates in vacuoles (Wu et al., 2022). Sulforaphane (SFN), allyl isothiocyanate (AITC), phenyl isothiocyanate (BITC), and phenethyl isothiocyanate (PEITC) are examples of common ITC forms (Chen et al., 2020). ITCs are known to exhibit various biological activities, including tumor inhibition, alleviation of diabetes symptoms, protection of nerve tissue, promotion of bacterial growth, and potent antioxidant effects. These properties make isothiocyanates highly promising for practical applications in the food industry, human health care, and agricultural production (Kiat et al., 2019; Maeda and Murakami, 2024; Wu et al., 2022). For instance, treatment with 50 μLL^-1^ IAITC significantly inhibits browning in *Flammulina velutipe*, preserves their structural integrity, reduces weight loss, and suppresses bacterial growth on the surface (Zhu et al., 2023). Treatment of mulberries with 5 μLL^-1^ and 15 μLL^-1^ of AITC at 5 °C effectively preserved higher levels of soluble solids and titratable acidity, inhibited respiration, maintained surface color and firmness, and extended the storage life of the fruit (Chen et al., 2015); treating blueberries with 5 μLL^-1^ AITC at 10°C effectively suppresses microbial growth and reproduction, thus delaying fruit spoilage (Wang et al., 2009); treatment of blackberries with 2 μLL^-1^ of AITC at 4°C and 80% relative humidity effectively preserved anthocyanin content, maintained surface color and physiological quality, and inhibited the development of gray mold (DoSu et al., 2023), and treatment with 5 μLL^-1^ of AITC effectively reduces the respiration rate and weight loss of fresh-cut purple cabbage, while maintaining its quality and nutritional value, thereby improving postharvest shelf life (Jiang et al., 2018); AITC treatment extends their shelf life of tomatoes, inhibiting microbial growth, and prolonging the flavor of tomatoes (Diego et al., 2024). Furthermore, BITC effectively inhibits the development of gray mold in strawberries and delays their natural spoilage (Sun et al., 2021). But research on ITCs in postharvest contexts is limited, particularly in relation to matsutake, a high-value fresh food crop. This study expands the understanding of ITCs’ biological mechanisms in postharvest preservation and provides a foundation for further exploration and promotion of matsutake mushroom postharvest management. Matsutake (*Tricholoma matsutake*), a highly prized edible mushroom, is susceptible to rapid postharvest deterioration due to oxidative stress and microbial activity. Isoamyl isothiocyanate (IAITC), a natural volatile compound, has shown potential in preserving perishable produce by modulating oxidative and antioxidative homeostasis. This study explores the efficacy of IAITC in maintaining the postharvest quality of matsutake by regulating reactive oxygen species (ROS) levels, enhancing antioxidant enzyme activity, and suppressing lipid peroxidation. The findings highlight IAITC as a promising eco-friendly strategy to extend the shelf life and commercial value of matsutake.

## 2 Materials and methods

### 2.1 Material and treatments

In Shaqiao town (E 101°03′-101°21′, N 25°02′-25°22′,1900-2680m), Nanhua County, Yunnan Province, fresh matsutake were hand-picked from a conservation forest and transferred to the College of Food Science and Technology lab at Yunnan Agricultural University in less than three hours below 8°C. Matsutakes of uniform size that were unharmed by microbiota or mechanical factors were chosen. To allow the volatile essential oil isoamyl isothiocyanate (IAITC, Shanghai Yuanye Bio-Technology Co., Ltd.) to volatilize, the matsutake were placed in a plastic sealed container lined with filter paper. The filter paper was then left at 25°C for 15 minutes. Distilled water served as the control, and the applied IAITC concentrations were 0, 10, 30, and 60 μLL^-1^, respectively. They were termed as groups of I, II, III and CK respectively. There were 15 matsutakes for each treatment. The containers were stored at 5.6 ± 0.6°C and 75 ± 5% relative humidity, and three replicates from each group were sampled at two days intervals for up to 8 days of storage.

### 2.2 Measurement of quality-related indices

#### 2.2.1 Measurement of weight loss, browning degree and firmness

The total weight difference between the matsutake before and after storage was used to calculate weight loss. The formula for calculating weight loss was *X=(W_0_−W_t_)/W_0_×100%*, where W_0_ and Wt stand for the mushroom’s starting weight and the weight of each sampling day, respectively. The L*, a* and b* values measured in the transverse section (Xiong et al., 2021) of matsutake were determined by a color difference meter (CM-5, KONICA MINOLTA). The matsutake fruiting body without the outer skin is cut into a cube of 1 cm^3^ for color difference measurement. The L* represents brightness, and the a* represents redness, b* indicates yellowness. The browning value is calculated by Equations 1, 2.


(1) Bi=100(x−0.31)/0.17

(2) X=(a+1.75L)/(5.546L+a−3.012b)

The firmness of matsutake was determined by instable Micro Systems Texture Analyzer (model TA-XT plus, SMS, England), using P/36R probe.

#### 2.2.2 Measurement of soluble proteins, soluble sugars, MDA, free amino acids and ASA contents

Matsutake was found to have free amino acids, MDA, soluble sugars, soluble proteins, and ASA levels were determined according to the method described by (Cao et al., 2007). The MDA concentration was reported on a fresh weight (FW) basis as nmol g^-1^., soluble sugar as the content (%) of soluble sugar on a fresh weight, and soluble proteins, ASA, and free amino acids as mg 100g^-1^ on a fresh weight (FW) basis.

#### 2.2.3 Measurement of total phenolics contents

The method described by (Shen, 2018) was used to determine the total phenolics contents. Grams of gallic acid equivalents (GAE) per gram of fresh weight were employed to express the phenolic contents, with gallic acid serving as a benchmark.

#### 2.2.4 Measurement of chitin contents

Using commercial kits (Jingmei; Nanjing, China), the chitin contents were calculated in accordance with the manufacturer’s instructions and reported as ngL^-1^.

### 2.3 Measurement of enzymes activities

To analyze the activities of the following enzymes, 0.5 g of matsutake samples were ground with 2 mL of pre-cooled phosphate buffer (50 mM, pH7.0), centrifuged 3000g, 4 °C, 10 min, and the crude enzyme extracts of polyphenol oxidase (PPO), peroxidase (POD), catalase (CAT), and superoxide dismutase (SOD) were extracted from the supernatant.

#### 2.3.1 PPO activity

The PPO activity was measured using the methodology outlined by (Yu, 2017). A change of 0.01/min in absorbance at A420 was one unit of PPO activity. Fresh weight, or FW−1, was used to express PPO activity.

#### 2.3.2 POD activity

POD activity was measured using the methodology outlined by (Yu, 2017). A change of 0.01/min in absorbance at A470 was one unit of POD activity. Fresh weight, or FW^−1^, was used to express POD activity.

#### 2.3.3 SOD activity

SOD activity assay as described by (Xue et al., 2023). One unit of SOD activity was defined as caused by a 50% inhibition of NBT. SOD activity was expressed as fresh weight, FW^−1^.

#### 2.3.4 CAT activity

CAT activity assays were performed using commercial kits (BOXBIO; Beijing, China) and in accordance with the manufacturer’s instructions. One unit of CAT activity was defined that Catalyzing the degradation of H_2_O_2_ at a rate of once per minute. CAT activity was expressed as fresh weight, FW−1.

### 2.4 Scanning electron microscopy

The day 0, day 4, and day 8 samples of Matsutake samples were fixed overnight in 2.5% glutaraldehyde solution at 4°C. The samples were then rinsed with PBS (100 mM, pH7.0) three times, each for 15 minutes. Afterward, the samples were fixed with 1% osmium tetroxide solution for 1–2 hours. The osmium waste solution was removed, and the samples were rinsed with PBS (100 mM, pH7.0) three times for 15 minutes each. Ethanol solutions with increasing concentrations (30%, 50%, 70%, 80%, 90%, and 95%, v/v) were then used to dry the samples. Each concentration was applied for 15 minutes, and then there were two treatments with 100% ethanol for 20 minutes each. A Leica EM CPD300 critical point dryer was then used to dry the samples at the critical point. Finally, the samples were coated and viewed with a Hitachi SU-8100 scanning electron microscope.

### 2.5 Statistical analysis

Every experiment was carried out three times, and the results were all expressed using the mean ± standard deviation (SD). To evaluate the experimental data, SPSS 26.0 (IBM Inc., Armonk, NY, USA) software was utilized. Statistical significance was assessed using Duncan’s test and one-way analysis of variance (ANOVA), with a significance threshold of P<0.05. Origin 2024 (Origin-Lab Co., Northampton, MA, USA) was used to visualize the data. Principal component analysis (PCA) was performed to compare the differences of each group.

## 3 Results

### 3.1 Changes of appearance and microstructure observation

**Figure 1:**
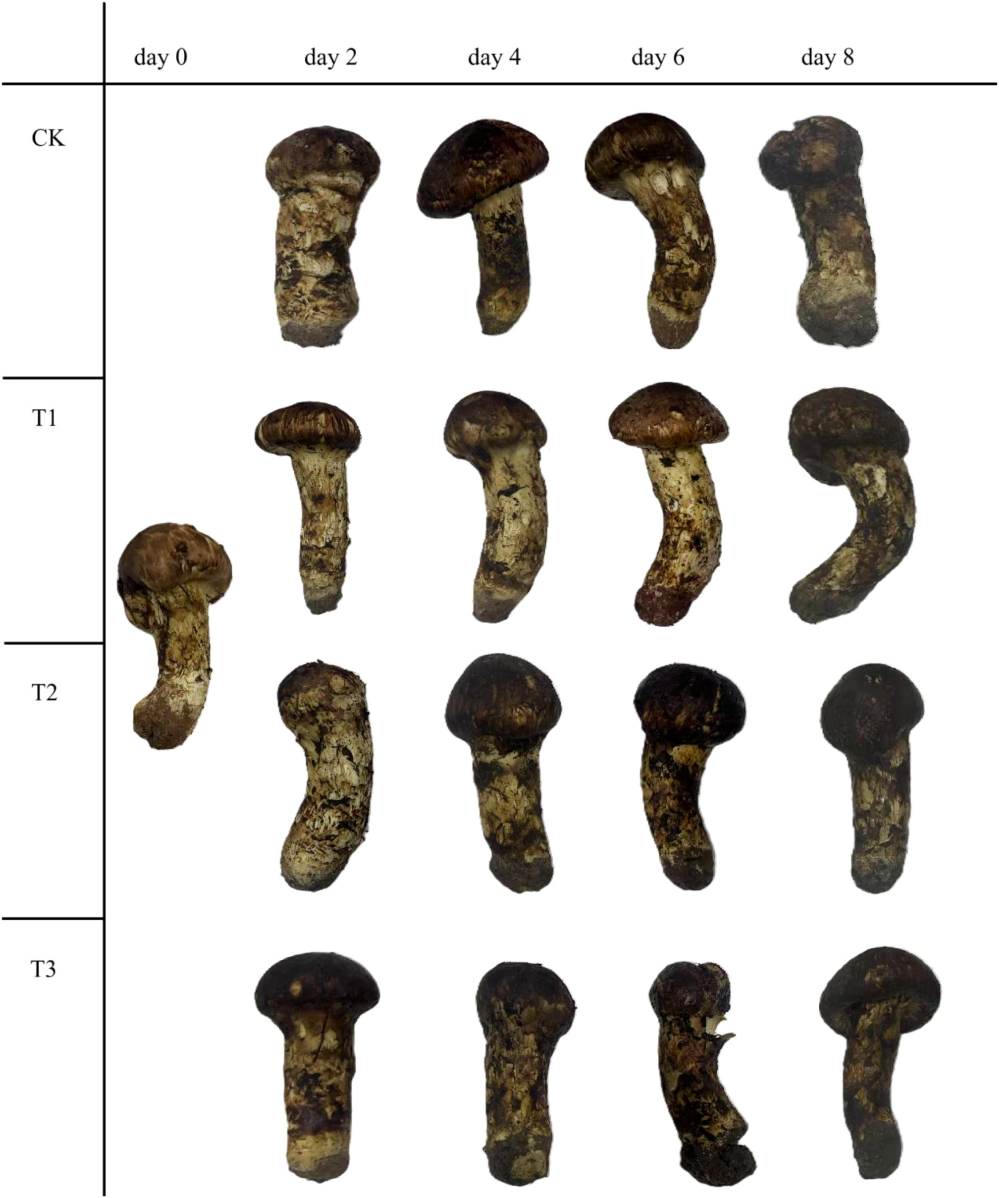
Gross appearance of matsutake with different treatment stored at 5.6 ± 0.5°C and 75 ± 5% relative humidity.

The appearance changes of matsutake during storage at 5.6 ± 0.6°C and 75 ± 5% relative humidity was shown in (**Figure 1**). In the control group (Eliezer et al.), the matsutake retained a color similar to that of the fresh samples for the first 4 days, after which the stem of matsutake began to turn black by day 6. In group I, the samples stored retained their appearance for the first 6 days, with the stem of matsutake beginning to turn black on day 8. In group II, the stem of matsutake began to turn black on day 4, and by day 8, the tissue exhibited water soaking. In group III, the stem of matsutake started to turn black on day 2, and by day 4, the tissue showed signs of water soaking.

**Figure 2:**
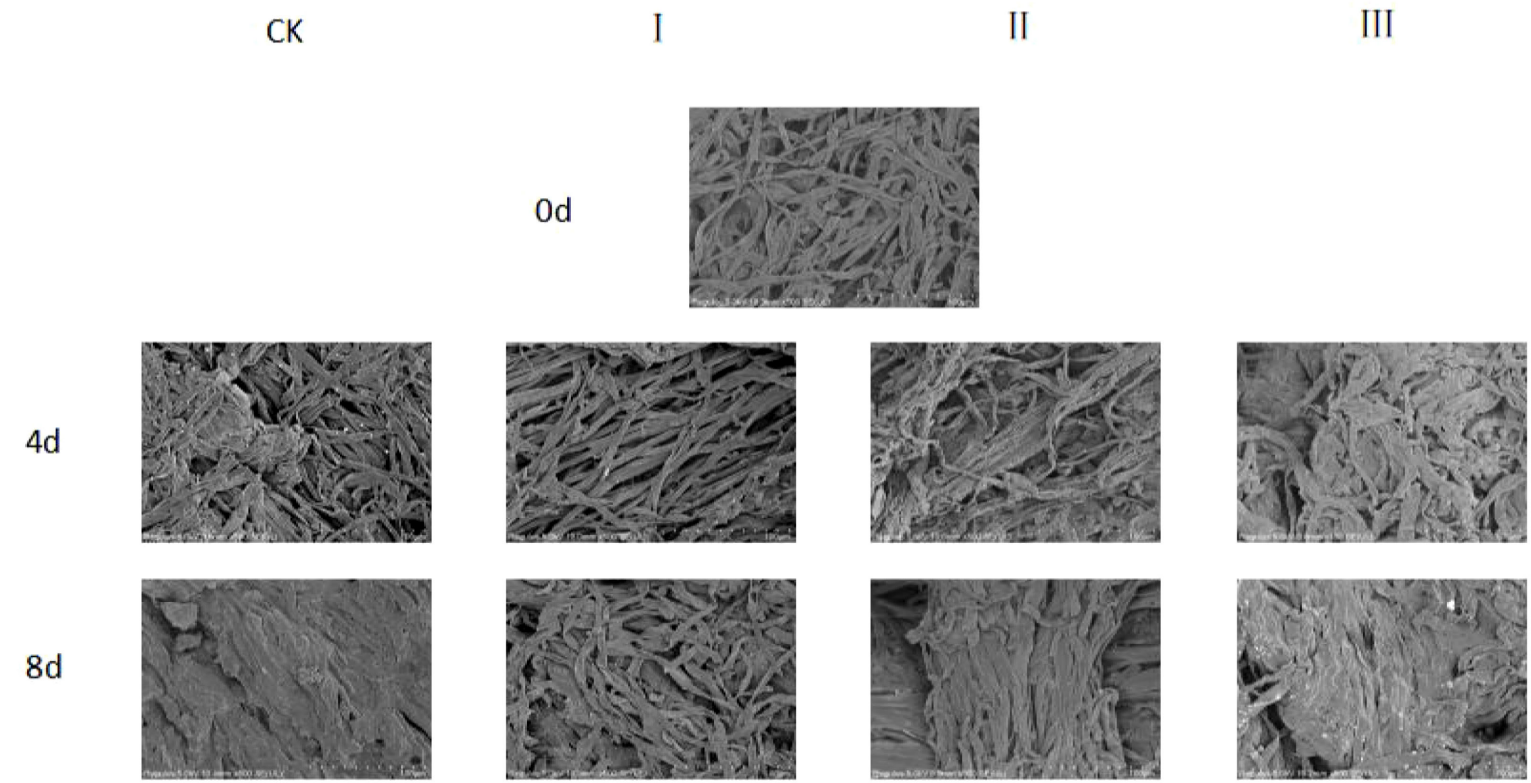
Microstructure of the shank appearance of matsutake under different treatments and times observed by SEM. (100 μm).

SEM observations of the stems of matsutake fruiting bodies revealed that on day 0, the structural textures of the stems were clearly distinguishable (**Figure 2**). By the 4^th^ day, the stem tissues of the CK, II, and III groups began to exhibit partially flattened (pancake-like) structures, whereas the stem textures of Group I remained relatively clear. By the end of the storage period, the stems of the CK group had completely transformed into flattened structures, and most stems in Groups II and III also displayed this morphology. In contrast, only parts of the stems in the Group I exhibited flattened structures.

### 3.2 Changes in weight loss, firmness and color

**Figure 3:**
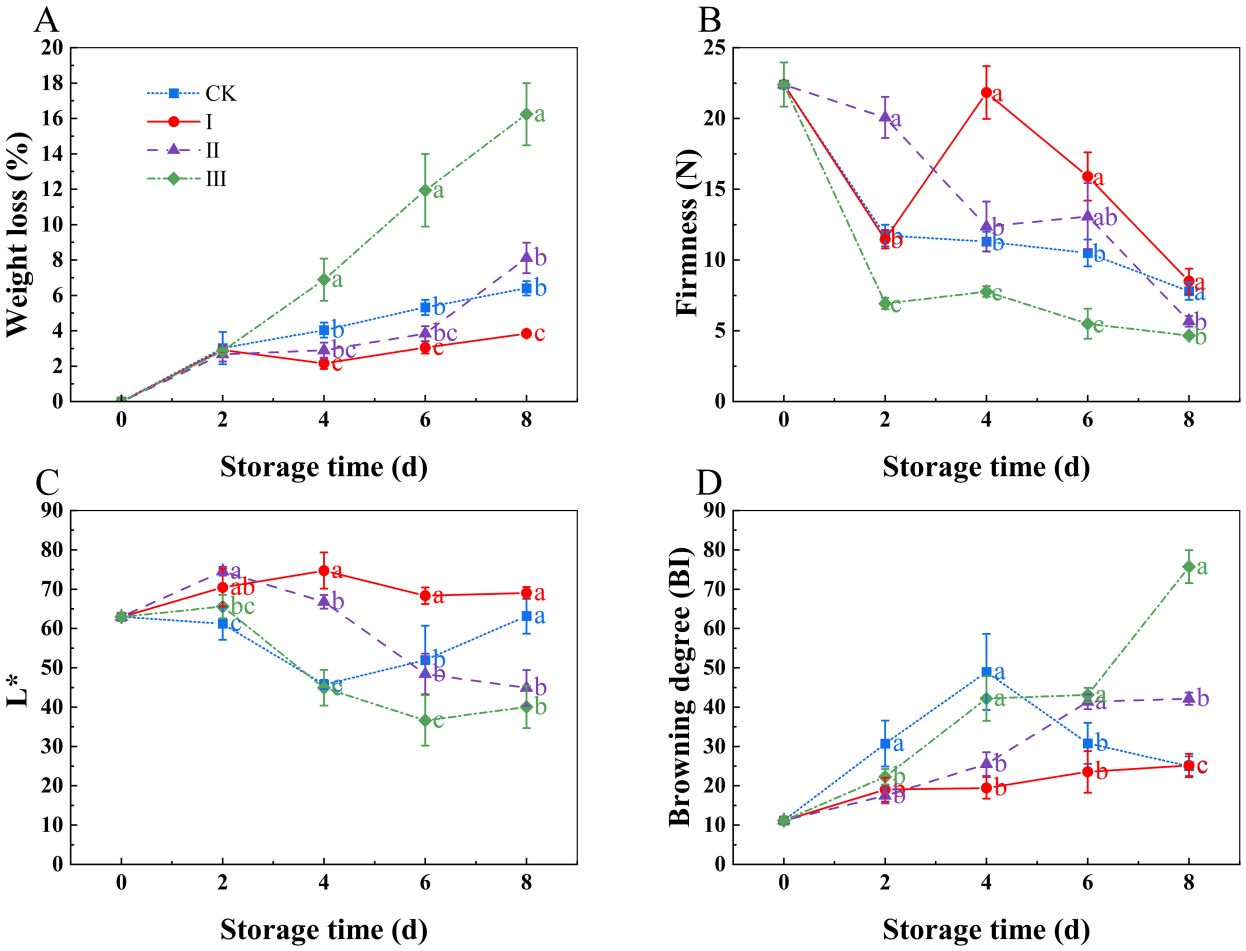
Effects of Isoamyl Isothiocyanate treatments on weight loss **(A)**, firmness **(B)**, L* **(C)**, browning degree **(D)** of matsutake. Error bars indicate SD calculated from three replicate experiments, and differing letters indicate significant (p<0.05) differences among different groups on the same day at storage time.

As the storage period increased, the weight loss rate showed an upward trend (**Figure 3A**). Starting day 6, group I experienced considerably less weight loss compared to the control group (Eliezer et al.) (*p*< 0.05). There was no significant difference between group II and the CK group (*p*<0.05). However, group III had a considerably higher weight loss rate than the other groups from day 4 (*p*<0.05). At the end of the 8-day storage period, the weight loss rates for the CK, I, II, and III groups were 6.4%, 3.84%, 8.12%, and 16.24%, respectively.

As storage time increased, the firmness of the matsutake decreased (**Figure 3B**). Group I’s firmness was much higher than that of the other groups (*p*<0.05); group II and the CK group showed no notable difference (*p*<0.05). Group III had the lowest firmness throughout the storage period. Over time, the Bi value rose whereas the L* value fell (**Figures 3C, D**). During the storage period, group I’s L* value was significantly greater than the CK groups, while group I’s Bi value stayed lower than the CK group’s (*p*<0.05).

### 3.3 Changes in nutrients and bioactive content

**Figure 4:**
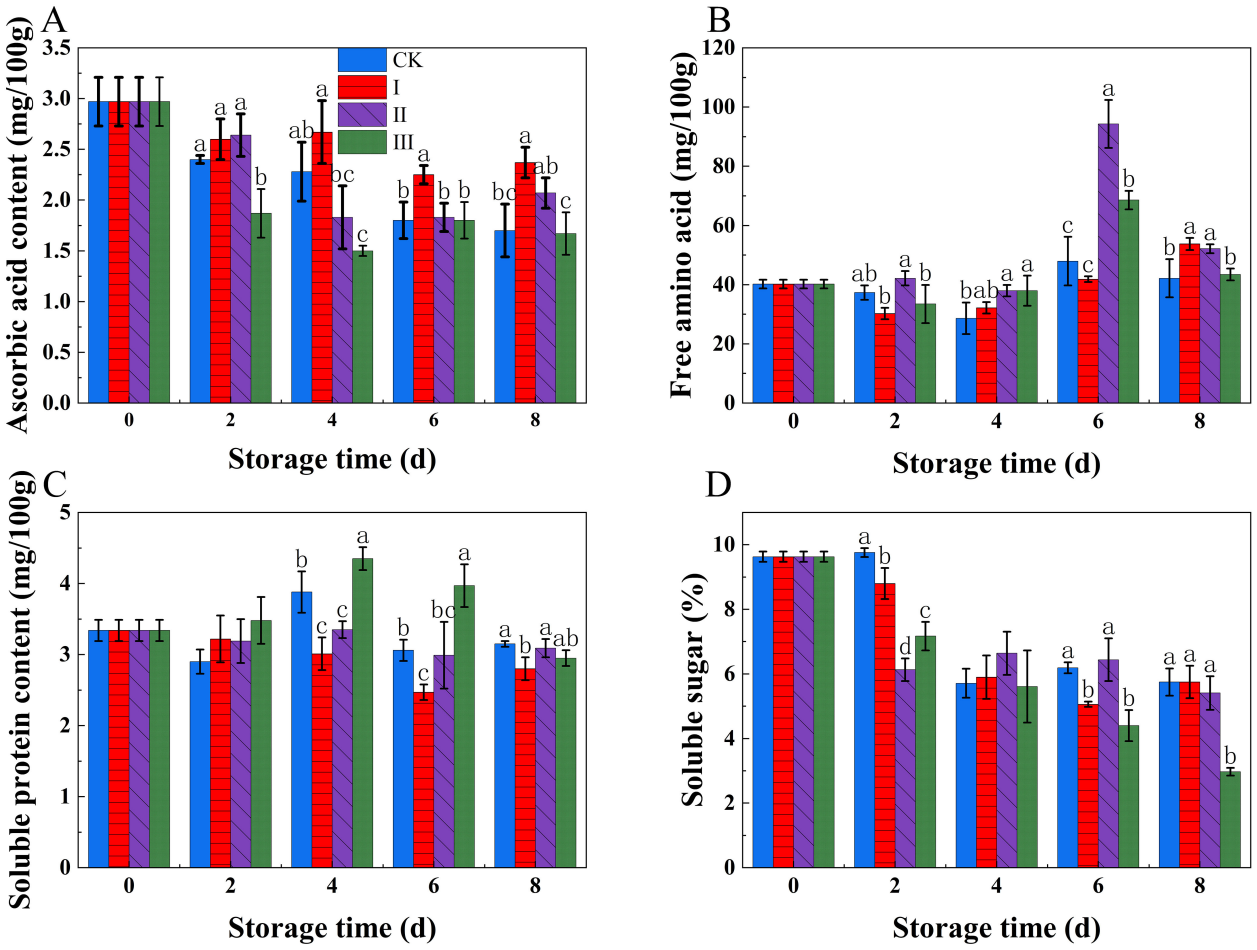
Effects of Isoamyl Isothiocyanate treatments on ASA content **(A)**, free amino acids **(B)**, soluble protein content **(C)**, soluble sugar **(D)** of matsutake. Error bars indicate SD calculated from three replicate experiments, and differing letters indicate significant (p<0.05) differences between groups for a given storage period.

As the storage period increased, both ASA and soluble sugars gradually decreased (**Figures 4A, D**). In group I, Throughout the storage time, the ASA material stayed much greater than in the other groups, maintaining a relatively high level (*p*<0.05). In contrast, group III had the lowest ASA content, and its levels of soluble sugars were much lower than those of the other groups (p<0.05).

During storage, soluble protein and free amino acids exhibited a rising and then declining trend (**Figures 4B, C**). Group I kept rather modest quantities of soluble protein and free amino acids. In contrast, group III showed considerably larger quantities of soluble protein (p<0.05) and free amino acids compared to the other groups.

**Figure 5:**
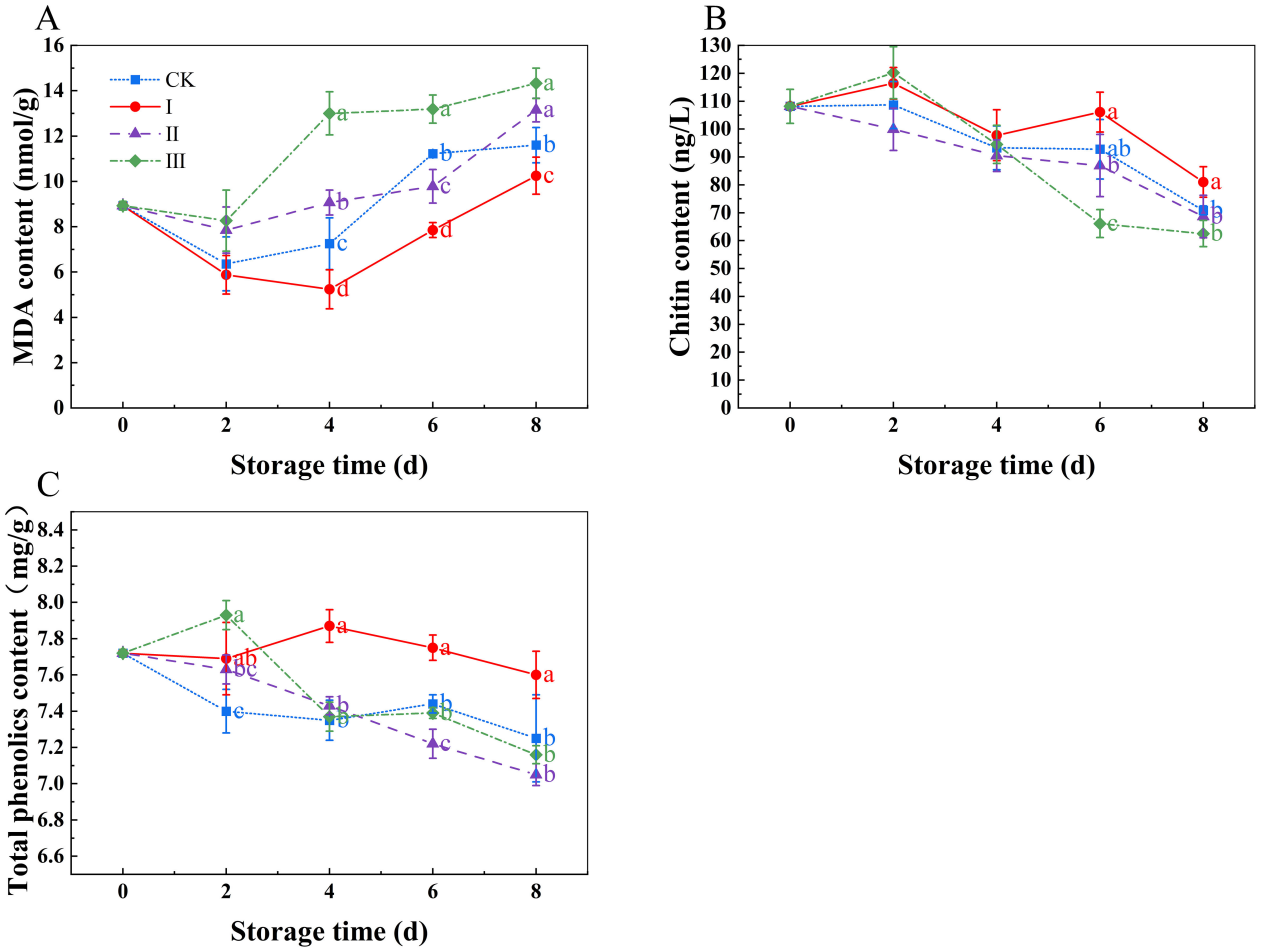
Effects of Isoamyl Isothiocyanate treatments on MDA content **(A)**, chitin content **(B)**, total phenolics content **(C)** of matsutake. Error bars indicate SD calculated from three replicate experiments, and differing letters indicate significant (p<0.05) differences between groups for a given storage period.

During storage, MDA content increased (**Figure 5A**), with significant differences beginning on day 4 (p<0.05). MDA levels were considerably lower in group I compared to the CK group (p<0.05), but significantly higher in group III (P<0.05). The MDA levels at the end of the storage period were I (10.25 nmol g^-1^), II (13.15 nmol g^-1^), III (14.33 nmol g^-1^), and CK (11.6 nmol g^-1^). The chitin and total phenol contents decreased during storage (**Figures 5B, C**), with group I show the slowest decline in both chitin and total phenolics, maintaining relatively high levels. In contrast, group III experienced the most rapid decline in both chitin and total phenolics, maintaining lower levels.

### 3.4 Changes in metabolic enzyme activity

**Figure 6:**
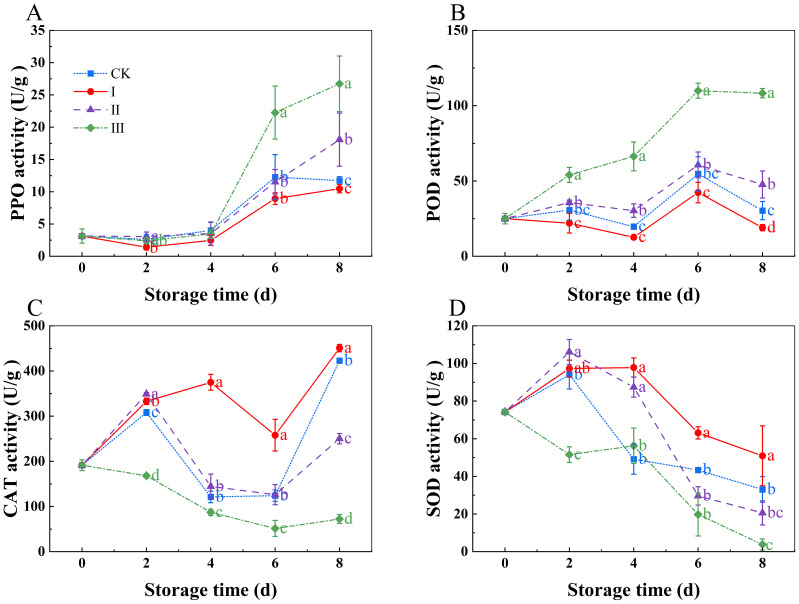
Effects of Isoamyl Isothiocyanate treatments on PPO activity **(A)**, POD activity **(B)**, CAT activity **(C)**, SOD activity **(D)** of matsutake. Error bars indicate SD calculated from three replicate experiments, and differing letters indicate significant (p<0.05) differences between groups for a given storage period.

PPO and POD activities rose with storage duration, as seen in (**Figures 6A, B**). Group III had significantly higher PPO and POD activity compared to other treatment groups (p<0.05), although group I had significantly lower levels (p<0.05). There were no noticeable differences between the CK group and group II. The CAT activity exhibited a rising-falling-rising trend with the storage time (**Figure 6C**). The CAT activity in group I was considerably higher than that in the CK group (*p*<0.05) and stayed high during the storage period. Group III, on the other hand, displayed the least amount of CAT activity during the storage period, greatly less than that of the other three treatment groups (*p*<0.05). SOD activity decreased with the storage time (**Figure 6D**). In group I, SOD activity decreased slowly throughout the storage period and remained significantly higher than that in the other groups (*p*<0.05). In group III, SOD activity remained at a low level throughout the storage period, approaching zero on day 8.

### 3.5 PCA analysis

**Figure 7:**
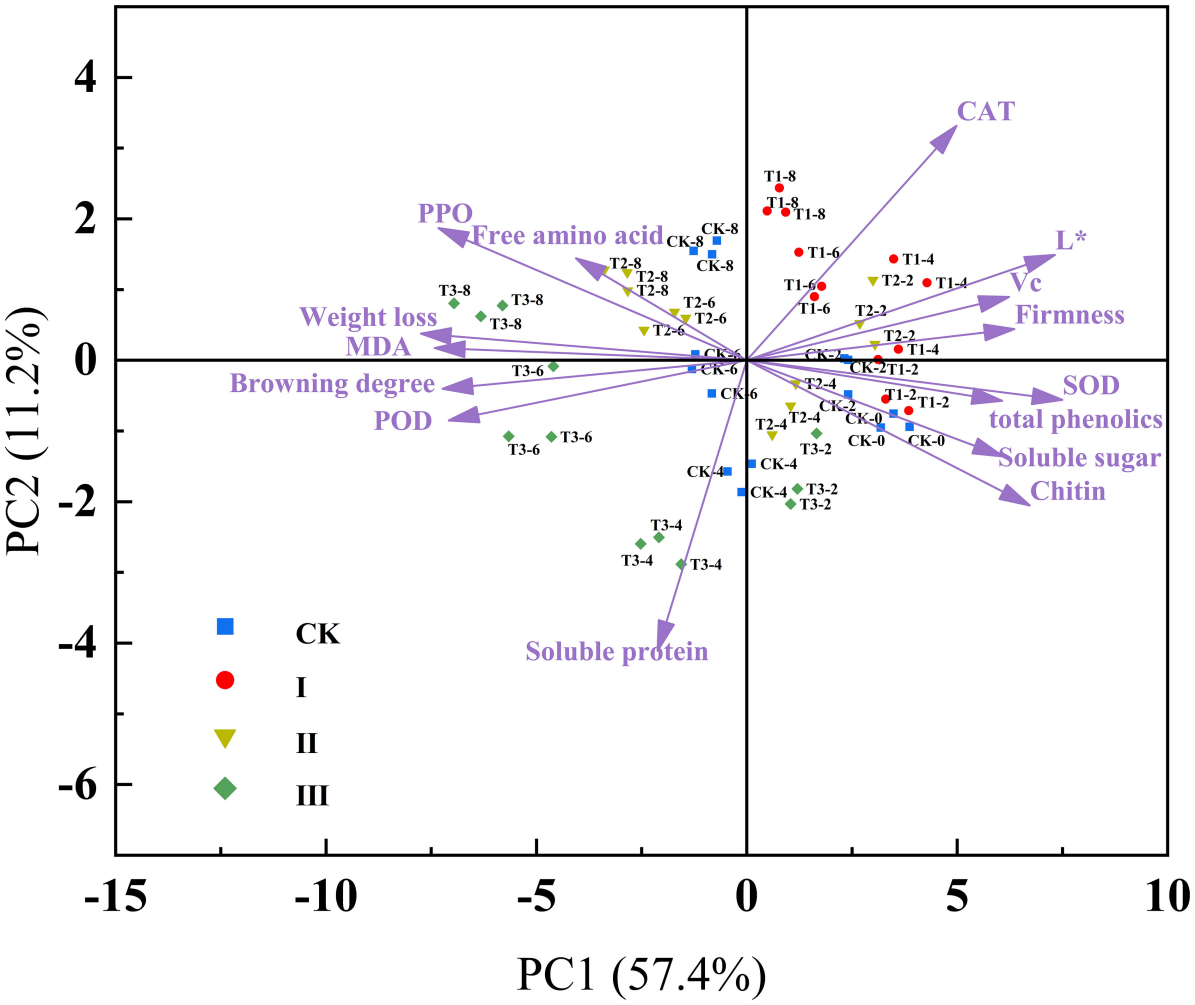
Principal component analysis score and loads plot of matsutake stored at 5.6 ± 0.5°C and 75 ± 5% relative humidity.

In order to show variables and evaluate how physiological quality markers vary over time during storage, PCA is an exploratory tool (Li et al., 2021). After principal component extraction (**Figure 7**), the cumulative variance contribution of PC1 and PC2 was 68.6%, indicating that most of the variations stemmed from these two principal components. Weight loss was positively correlated with MDA, POD, PPO, and browning degree. Firmness showed positive correlations with CAT, L*, ASA, SOD, total phenolics, soluble sugar, and chitin. The projections of L*, CAT, ASA, SOD, firmness, and phenolics on PC1 were relatively large, suggesting a strong correlation between these indicators and PC1. PPO, POD, MDA, weight loss, and browning degree were strongly positively correlated with PC2, indicating their secondary but important role in the preservation effect. Group I is located on the right side of PC1, which suggests that the preservative concentration in Group I has a better preservation effect, maintaining the stability of various quality indicators and indicating that the samples are in a fresher state. The samples from Group CK, from day 0 to 2, are closely grouped on the right side of the graph. However, from day 4 to 8 of storage, the samples shift to the left, reflecting changes in the samples, which align with the previously mentioned results.

## 4 Discussion

Currently, the chemical preservation of mushrooms predominantly involves the use of edible coatings (Liu, 2020). However, these materials degrade slowly and typically require the addition of other compounds to enhance their preservative effects. In contrast, allyl isothiocyanate (IAITC), a type of isothiocyanate (ITC) derived from cruciferous plants, has attracted considerable interest in food science due to its natural origin, biodegradability, relative safety, and antimicrobial properties (Zhu et al., 2023). Matsutake (*Tricholoma matsutake*) is a highly valued edible mushroom with a short shelf-life due to rapid postharvest deterioration, primarily caused by oxidative stress (Christiana et al., 2025). Isoamyl isothiocyanate (IAITC), a natural volatile compound, has shown efficacy in extending the postharvest quality of matsutake by regulating oxidative and antioxidative homeostasis. There are some keys mechanisms of IAITC action (Afzal et al., 2023); reduction of oxidative stress through IAITC suppresses the accumulation of reactive oxygen species (ROS), such as superoxide anion (O_2_-) and hydrogen peroxide (H_2_O_2_), which are major contributors to cellular damage in postharvest mushrooms. As well as, by inhibiting lipid peroxidation (measured via malondialdehyde, MDA), IAITC helps maintain membrane integrity, preventing cell leakage and senescence. The other was enhancement of antioxidant defense systems through IAITC upregulates the activity of key antioxidant enzymes, including superoxide dismutase (SOD), catalase (CAT), and peroxidase (POD), which scavenge harmful ROS. And it also boosts non-enzymatic antioxidants (e.g., glutathione, GSH), further enhancing cellular resistance to oxidative damage. The last was delayed senescence and quality preservation by balancing oxidative and antioxidative processes, IAITC slows down protein degradation, reduces browning, and maintains firmness, color, and nutritional quality. This modulation helps extend the commercial shelf-life of matsutake while preserving its unique aroma and texture. IAITC treatment offers a promising natural strategy for postharvest preservation of matsutake by maintaining redox balance. Further research could explore its application in other perishable fungi and optimal treatment conditions for industrial use. Since buyers frequently use color as the main criterion to evaluate quality without direct contact, the color of mushrooms is a significant indicator of market value (Lei et al., 2018). The results of our investigation indicated that the matsutake treated with 10 μLL^-1^ IAITC exhibited the best performance throughout the storage period, particularly in terms of weight loss, color, and firmness. The possible reason for this could be that IAITC reduced the consumption of organic substances (ASA, soluble protein, free amino acids, total phenolics etc.) in matsutake, thereby maintaining the chitin content and preserving the integrity of the cell membrane, which helped maintain the hardness of the matsutake. Additionally, IAITC inhibited the enzymatic browning (PPO) in matsutake, thereby preserving their color. On the other hand, 30 or 60 µLL^-1^ concentrations may have caused damage to the matsutake, leading to the continuous degradation of organic substances to sustain life activities, damage to the cell membranes, and exacerbation of browning in the matsutake.

The soluble protein, a key nutrient and osmotic regulator, is also an essential component of metabolic enzymes and plays a critical role in regulating various physiological activities. Its content level reflects the state of the organism’s tissues (Wang et al., 2017). As we can be seen from the results, the early-stage treatment with IAITC likely inhibited the physiological metabolic activities of the fruiting bodies, leading to reduced nutrient loss and maintaining soluble protein content at a relatively low level. During the middle stage of storage, the quality of the fruiting bodies gradually declined, and the permeability of the cell membrane increased, causing the outflow of soluble proteins from within the cells (Li et al., 2019b). This resulted in a slight increase in soluble protein content during the later stages of storage. Free amino acids are the main source of umami and aroma in matsutake mushrooms, and they are closely related to the unique flavor of matsutake. The changes in free amino acid content during the growth, development, maturation, and aging of matsutake mushrooms are closely associated with physiological and biochemical metabolism. With the increase in storage time, the free amino acids and soluble protein content in all experimental groups increased, especially with a significant increase in soluble protein at 30 or 60 µLL^-1^. This could be due to the increased permeability of the cell membrane, which causes the leakage of soluble proteins from within the cells. As the soluble protein content increased, the free amino acids content also increased. The soluble protein in matsutake degrade into free amino acids as storage time progresses, but free amino acids can also be oxidized into quinone-like substances, which leads to browning and changes in the umami and aroma of matsutake (Liu, 2020). The soluble protein and free amino acids content remained stable throughout the storage period at 10 µLL^-1^, while free amino acids at 30 or 60 µLL^-1^ began to boost from the sixth day, leading to an increase in the products of browning and further exacerbating the browning of matsutake.

Fungal cell walls are mostly composed of chitin, an unbranched polymer that is primarily composed of β-1,4-N-acetylglucosamine. Chitin is essential for preserving the structure of fungal cell walls. The higher the chitin content, the greater the rigidity of the hyphal cell wall (Munro, 2013). With the continued extension of storage time, matsutake mushrooms gradually become softer, and their chitin content shows a decreasing trend. The higher the chitin content, the more intact the cell membrane remains. As observed through scanning electron microscopy, matsutake mushrooms treated with 10 µLL^-1^ isothiocyanate retained a relatively good morphology, indicating that this treatment effectively maintained chitin content and preserved the normal morphology of the matsutake. The possible reason for this is that it enhanced the antioxidant activity within the matsutake mushrooms, thereby slowing down the accumulation of MDA, maintaining cell membrane integrity, and preserving the hardness of the matsutake.

The experimental results showed that as the storage period increased, the activities of PPO and POD also increased, and the degree of browning intensified. This is because phenolic compounds are oxidized into quinones under the action of enzymes, and these quinones further polymerize to form brown, yellow, or black polymers, which lead to browning in matsutake tissues (Yang et al., 2009). Browning occurs when PPO and POD oxidize phenolic compounds, leading to the formation of brown substances. PPO and POD work synergistically in the formation of brown polymers (Gao et al., 2017, 2014). The main reason of matsutake browning is the oxidation of phenolic compounds by PPO (Kiat et al., 2019; Li et al., 2019a), and the activity of PPO directly correlates with the intensity of the browning reaction. Our research found matsutake treated with 10 μLL^-1^ IAITC inhibited the activity of both PPO and POD, while maintaining the content of total phenolics, thereby reducing the oxidation of phenolics compounds and the production of secondary metabolites. This treatment also stabilized the free amino acids content, reducing the substrates for browning and maintaining the stability of matsutake color. On the other hand, at 30 or 60 µL L^-1^, PPO activity increased rapidly during storage. This could be due to oxidative damage to the matsutake, which caused an increase in PPO activity, as well as an increase in free amino acids content and a reduction in total phenolics. These changes led to an increase in the substrates for enzymatic browning, which exacerbated the browning and aging of matsutake mushrooms.

Reactive oxygen species (ROS) are involved in various metabolic processes in plant cells, and they can be toxic, causing oxidative damage and altering cell structure and function (Gill and Tuteja, 2010). Superoxide, hydrogen peroxide, and hydroxyl radicals are examples of ROS that can build up and cause tissue damage in plants, which lowers the quality of fruits and vegetables and lowers their market value (Hodges et al., 2004). SOD and CAT help mitigate the harmful effects of ROS (Yan et al., 2020). A strong and water-soluble antioxidant, ASA can prevent or lessen ROS-induced deterioration in fruits and vegetable (Nasiri et al., 2018). A report by (Sharma et al., 2019). demonstrated that total phenolics compounds possess the potential to scavenge harmful ROS and enhance tissue antioxidant capacity. Damage to the integrity of the cell membrane and the structure of the cell wall is reflected in the accumulation of MDA, a hallmark of membrane lipid peroxidation (Yang et al., 2022). Our study indicated matsutake treated with 10 μLL^-1^ IAITC maintained high levels of SOD and CAT activity, likely due to the upregulation of certain genes related to these enzymes by IAITC. By maintaining high SOD and CAT activity, IAITC reduced the ROS levels within the matsutake, thereby preserving the content of ASA and total phenolics. IAITC alleviated the ROS-induced damage to the cell membrane by maintaining antioxidant enzyme activity and antioxidant content, which in turn reduced MDA accumulation and preserved the normal structure of the matsutake cell membrane, the firmness of the matsutake was maintained, which helped delay aging. This finding is consistent with studies on mulberries (Chen et al., 2015), blueberries (Wang et al., 2009), purple cabbages (Jiang et al., 2018) and *Flammulina velutipes* (Zhu et al., 2023).

Through PCA visualization of the variable evaluation, we found that the weight loss rate, browning, and MDA, PPO, POD, and free amino acids showed positive correlations. Firmness was negatively correlated with MDA, weight loss rate, and browning, which is consistent with the previous discussion. The accumulation of MDA disrupted the integrity and structure of the cell membrane, leading to the softening of matsutake mushrooms, increased weight loss, and a water-soaked state in the tissue. The increase in PPO, POD, and free amino acids enhanced the activity of the enzymes responsible for enzymatic browning and increased the substrates, leading to the browning of matsutake. Nevertheless, firmness showed a positive correlation with antioxidant capacity (SOD, CAT, ASA, total phenolics), which also aligns with the previous discussion. This is because the treatment with 10 µLL^-1^ IAITC improved the antioxidant capacity of matsutake, reduced oxidative damage, and maintained the levels of organic compounds such as ASA, total phenolics, and chitin content. This helped preserve cell integrity, reduce weight loss, and inhibit PPO activity, thus delaying the browning and aging process.

To summarize, the treatment with 10 μLL^-1^ effectively preserves the original brightness of matsutake, reduces browning, mitigates quality loss, enhances antioxidant enzyme activity, and maintains antioxidant content. No significant differences were observed between the 30 μLL^-1^ treatment and the control group. However, the 60 μLL^-1^ treatment led to an increase in PPO activity and MDA content, resulting in a dull appearance and deterioration of the samples by the 4th day. These findings imply that excessive application of IAITC accelerates browning and aging processes. The 10 μLL^-1^ treatment may exert a preservative effect by activating the self-defense mechanisms of matsutake. This treatment significantly enhanced the activities of resistance-related enzymes, including CAT and SOD, resulting in increased levels of antioxidant substances, such as ASA and total phenolics, thereby improving the antioxidant capacity. However, to further validate these findings, additional research is needed to examine a range of ROS metabolism indicators, including changes in free radicals. The above conclusion indicates that a 10 μLL^-1^ treatment can effectively preserve matsutake, maintaining their nutritional components and enhancing antioxidant capacity. However, excessive application of IAITC disrupts the physiological metabolic processes of matsutake, leading to metabolic disorders, accelerating aging, and resulting in negative effects.

## 5 Conclusion

Isoamyl isothiocyanate (IAITC) effectively preserves the postharvest quality of matsutake (*Tricholoma matsutake*) by maintaining oxidative and antioxidative homeostasis. Through the suppression of reactive oxygen species (ROS) accumulation and lipid peroxidation, IAITC mitigates oxidative stress, thereby delaying cellular damage and senescence. Additionally, it enhances the activity of key antioxidant enzymes (SOD, CAT) and non-enzymatic antioxidants (e.g., glutathione), reinforcing the mushroom’s defense mechanisms. As a result, IAITC treatment helps retain the firmness, color, aroma, and nutritional value of matsutake, extending its shelf life. These findings highlight IAITC as a promising natural preservative for postharvest mushrooms, offering potential applications in food storage and commercial preservation technologies. Further studies could optimize treatment conditions and explore its efficacy in other perishable fungi.

## Data Availability

The original contributions presented in the study are included in the article/supplementary material. Further inquiries can be directed to the corresponding authors.
