# Supplementary figures and images for: Isoamyl isothiocyanate preserves postharvest quality of matsutake (Tricholoma matsutake) by modulating oxidative and antioxidative homeostasis
Source: Front Plant Sci. 2025 Jul 22;16:1627772. doi: 10.3389/fpls.2025.1627772 (PMC12322898; doi:10.3389/fpls.2025.1627772)

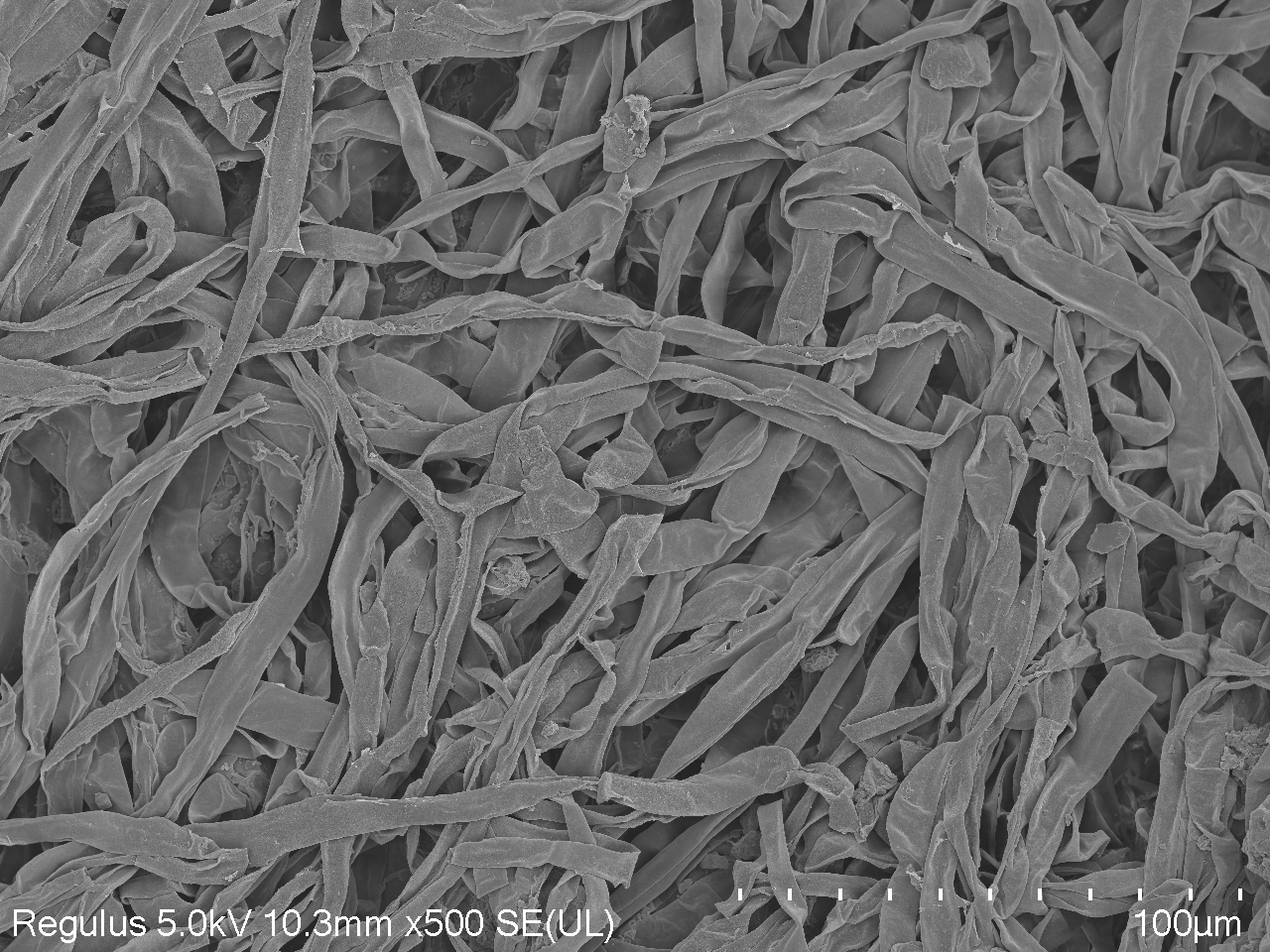

Supplement: Supplementary file 1 [file DataSheet1.zip › SEM/CK(day0).tif]

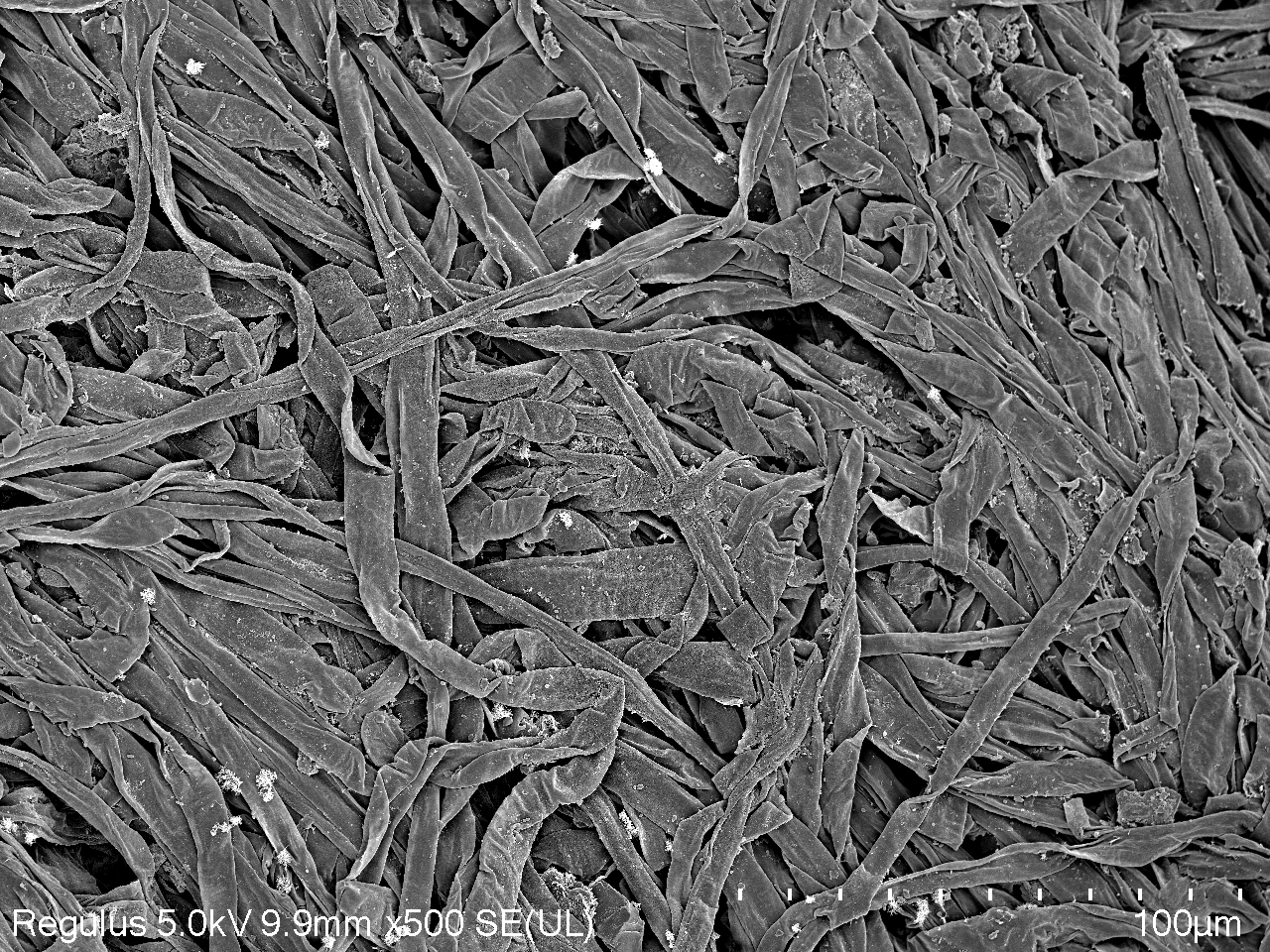

Supplement: Supplementary file 1 [file DataSheet1.zip › SEM/CK(day4).tif]

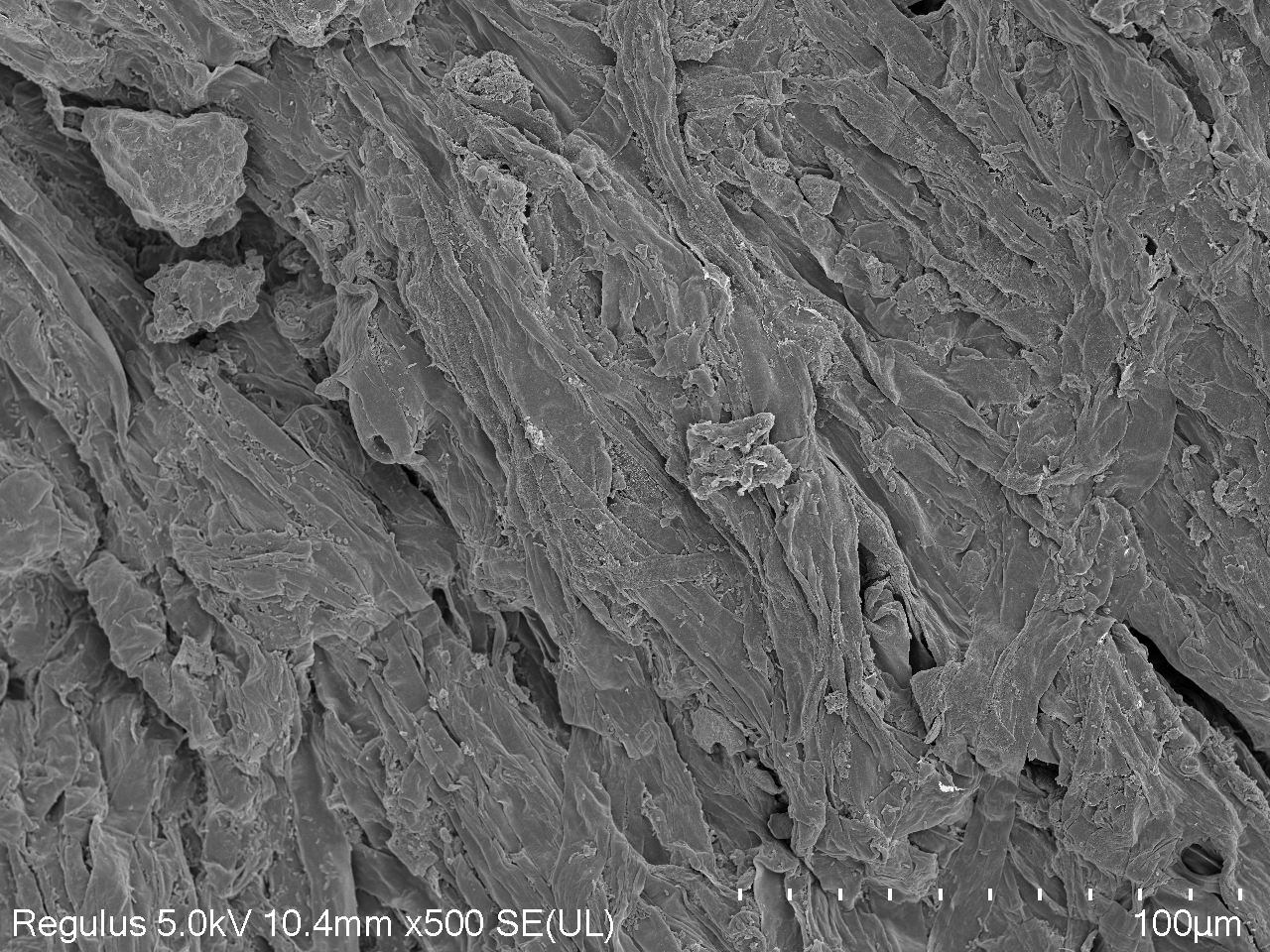

Supplement: Supplementary file 1 [file DataSheet1.zip › SEM/CK(day8).tif]

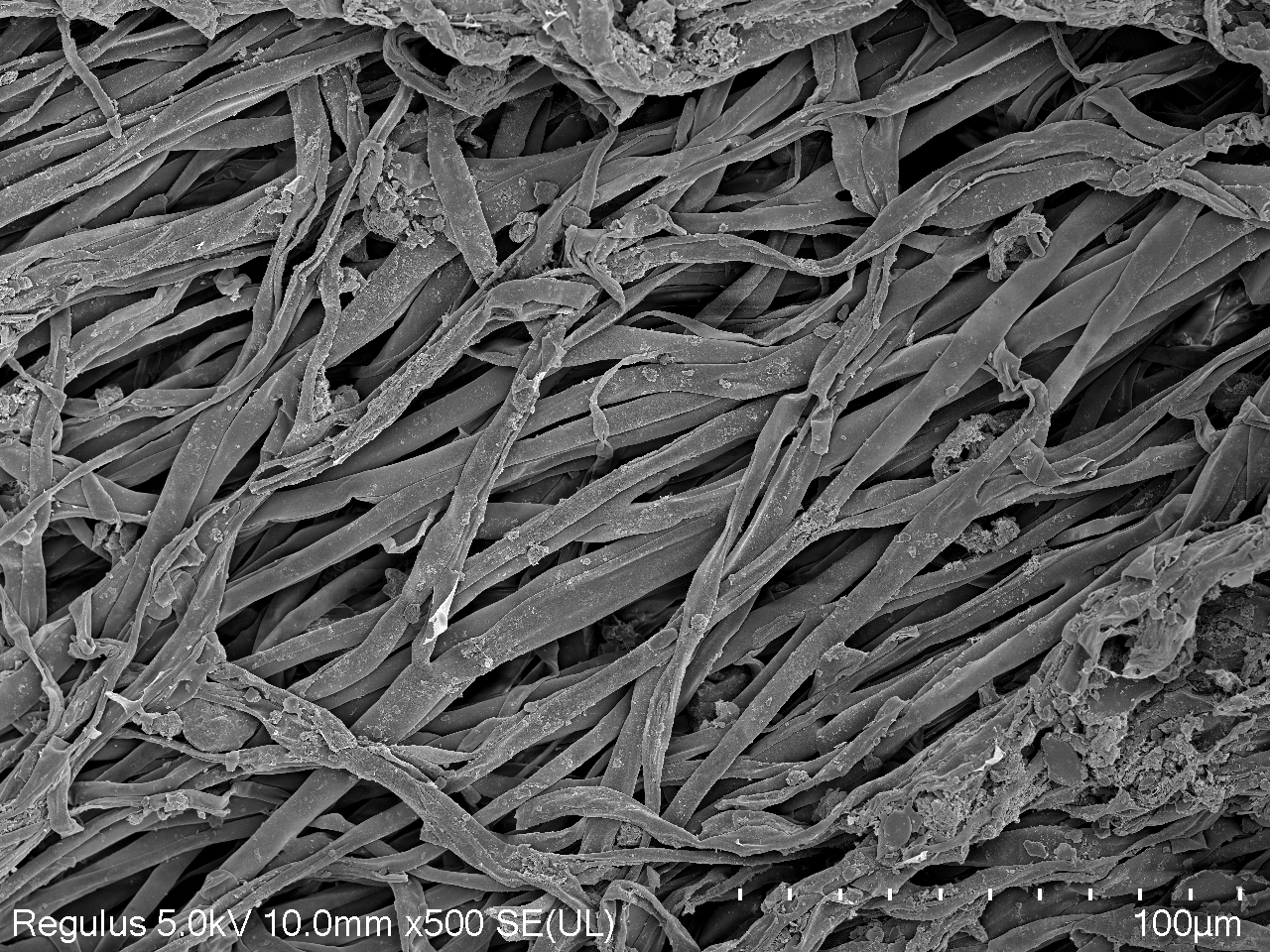

Supplement: Supplementary file 1 [file DataSheet1.zip › SEM/I(day4).tif]

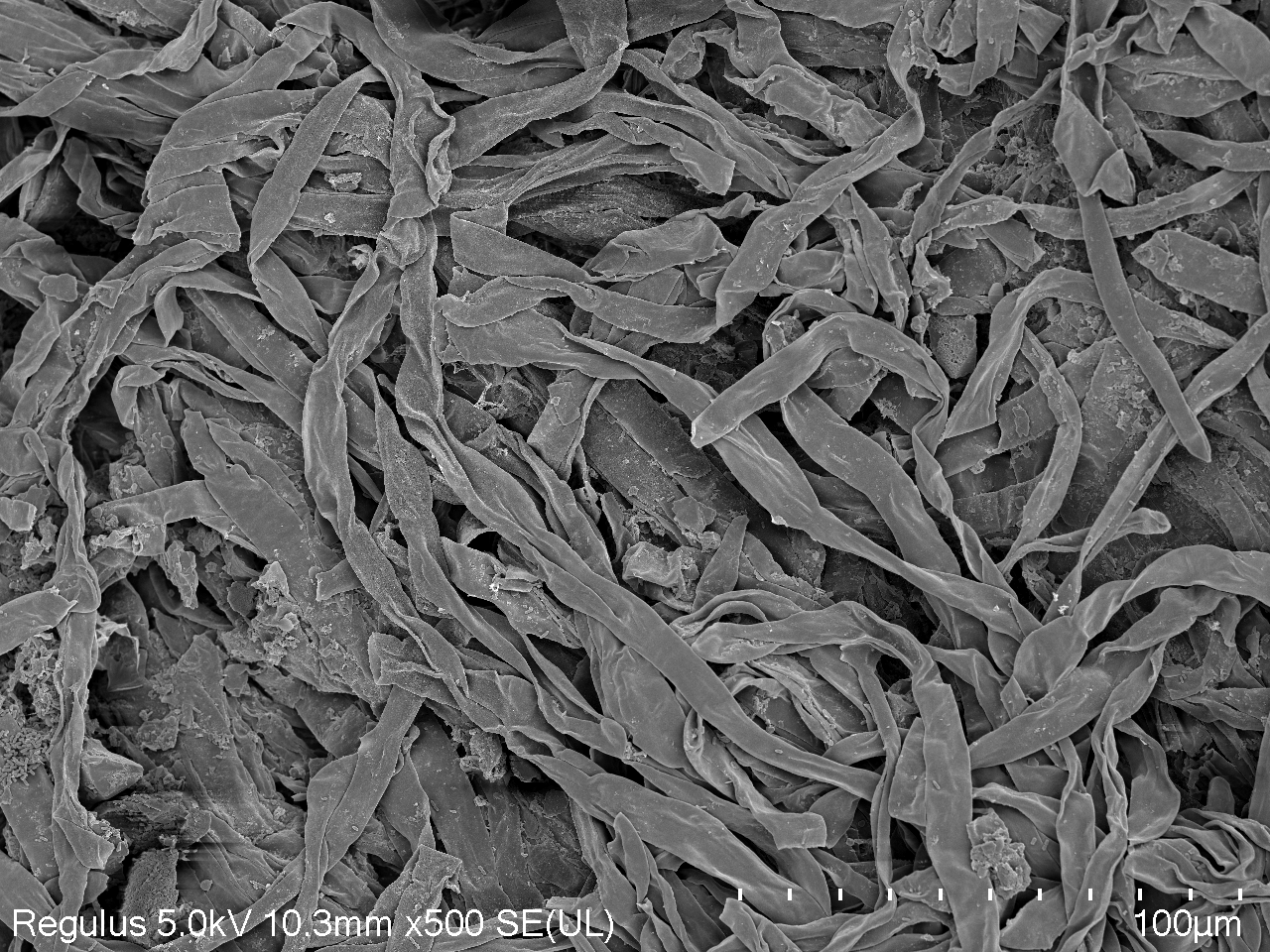

Supplement: Supplementary file 1 [file DataSheet1.zip › SEM/I(day8).tif]

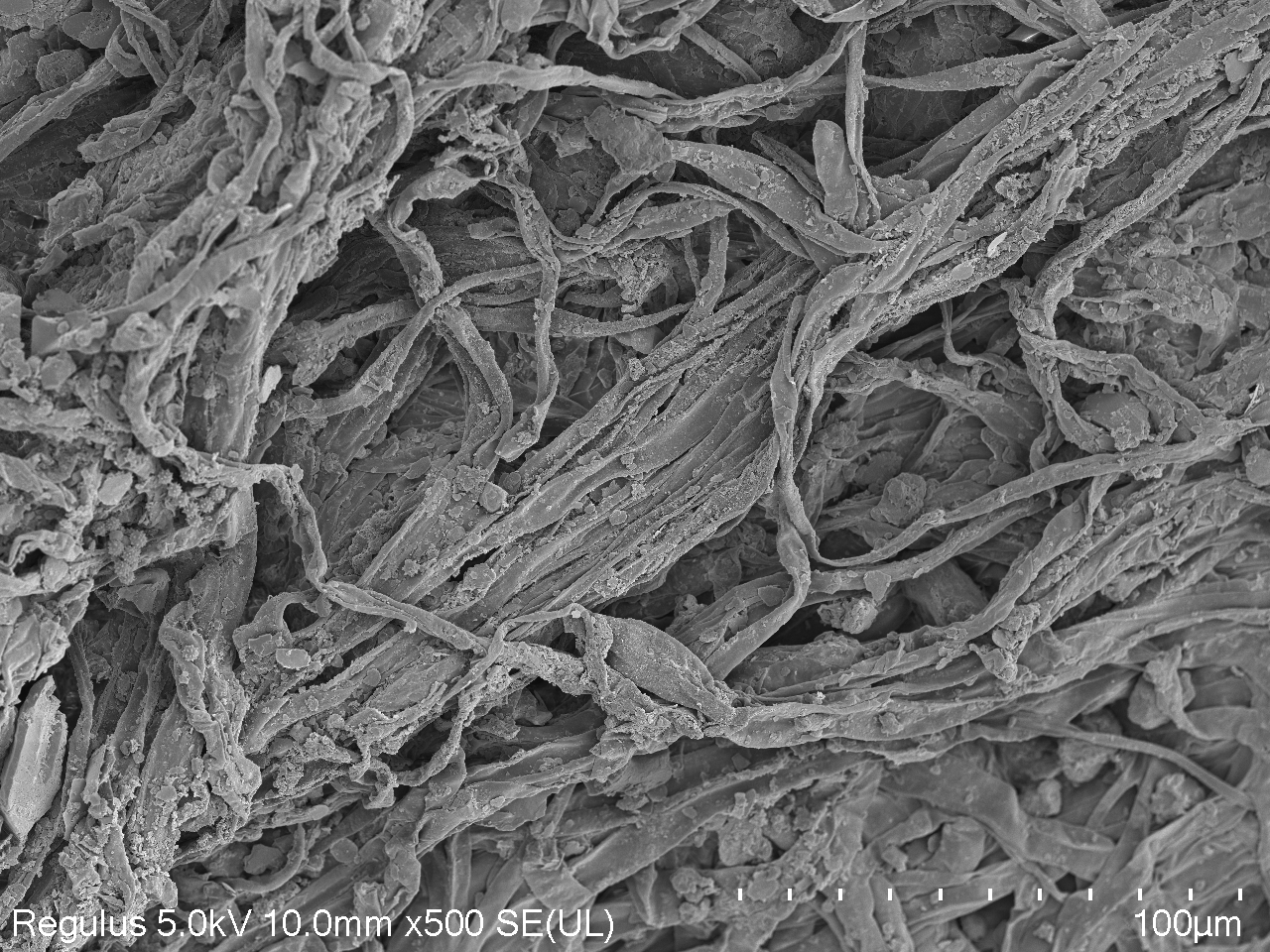

Supplement: Supplementary file 1 [file DataSheet1.zip › SEM/II(day4).tif]

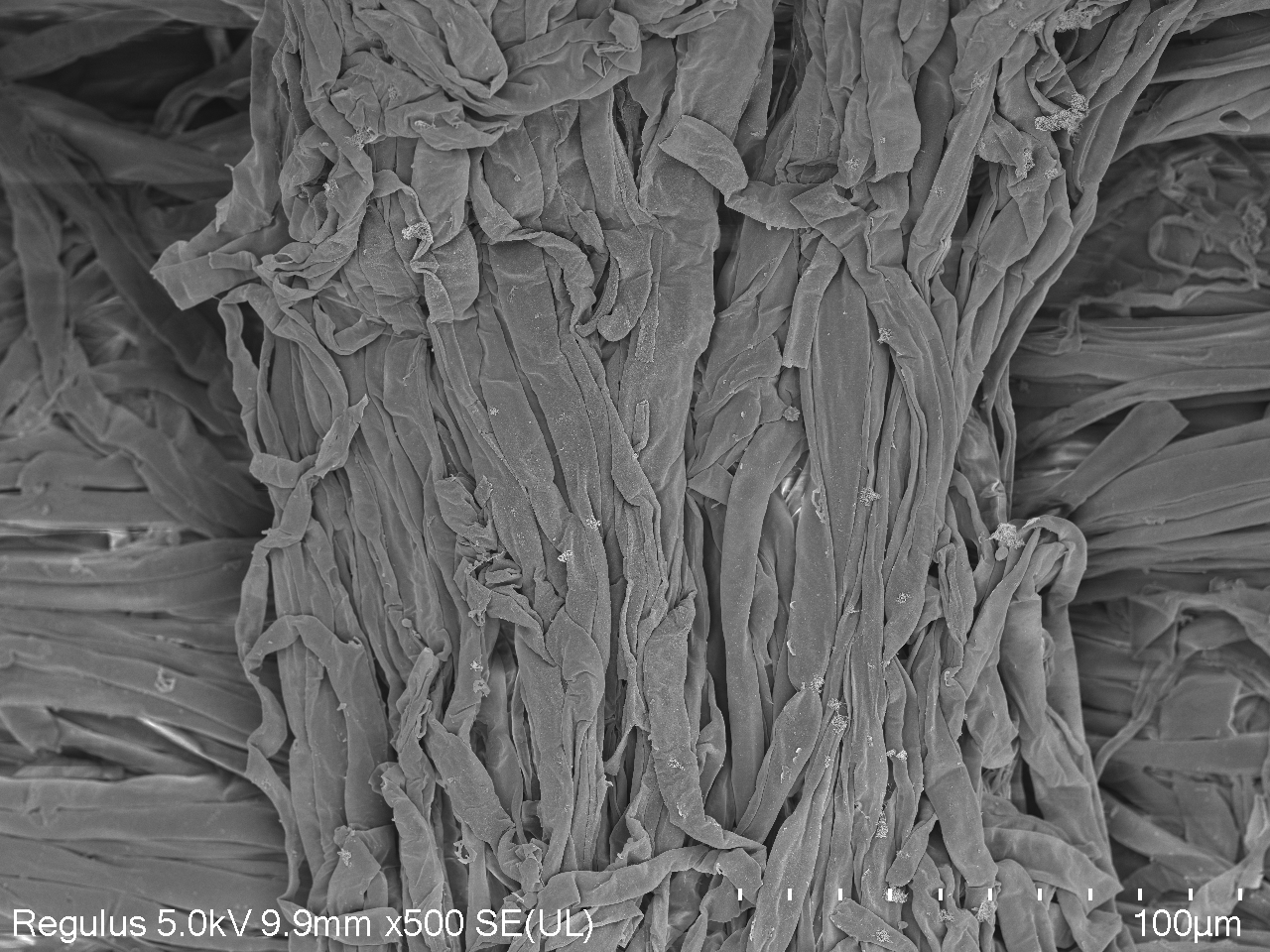

Supplement: Supplementary file 1 [file DataSheet1.zip › SEM/II(day8).tif]

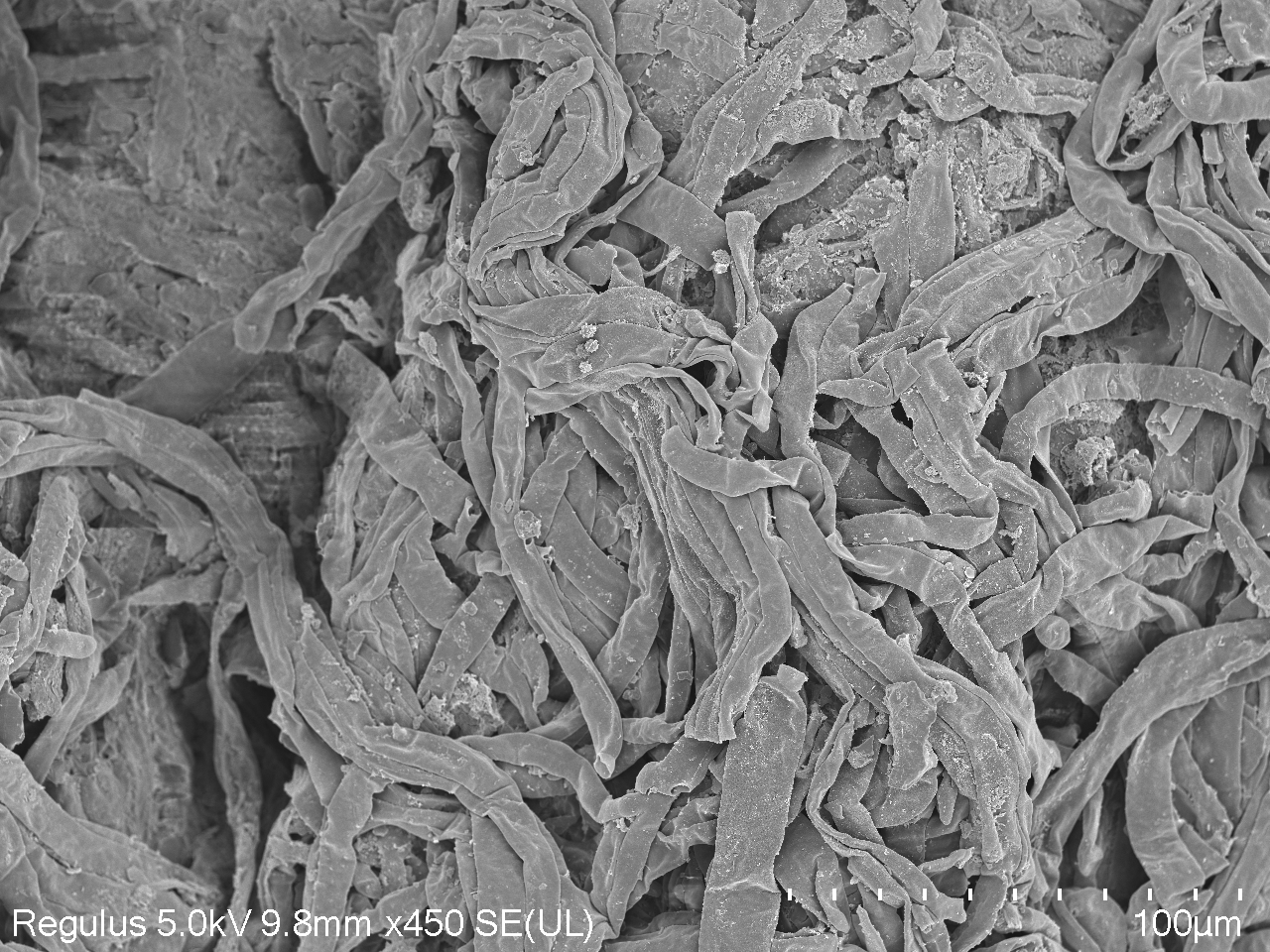

Supplement: Supplementary file 1 [file DataSheet1.zip › SEM/III(day4).tif]

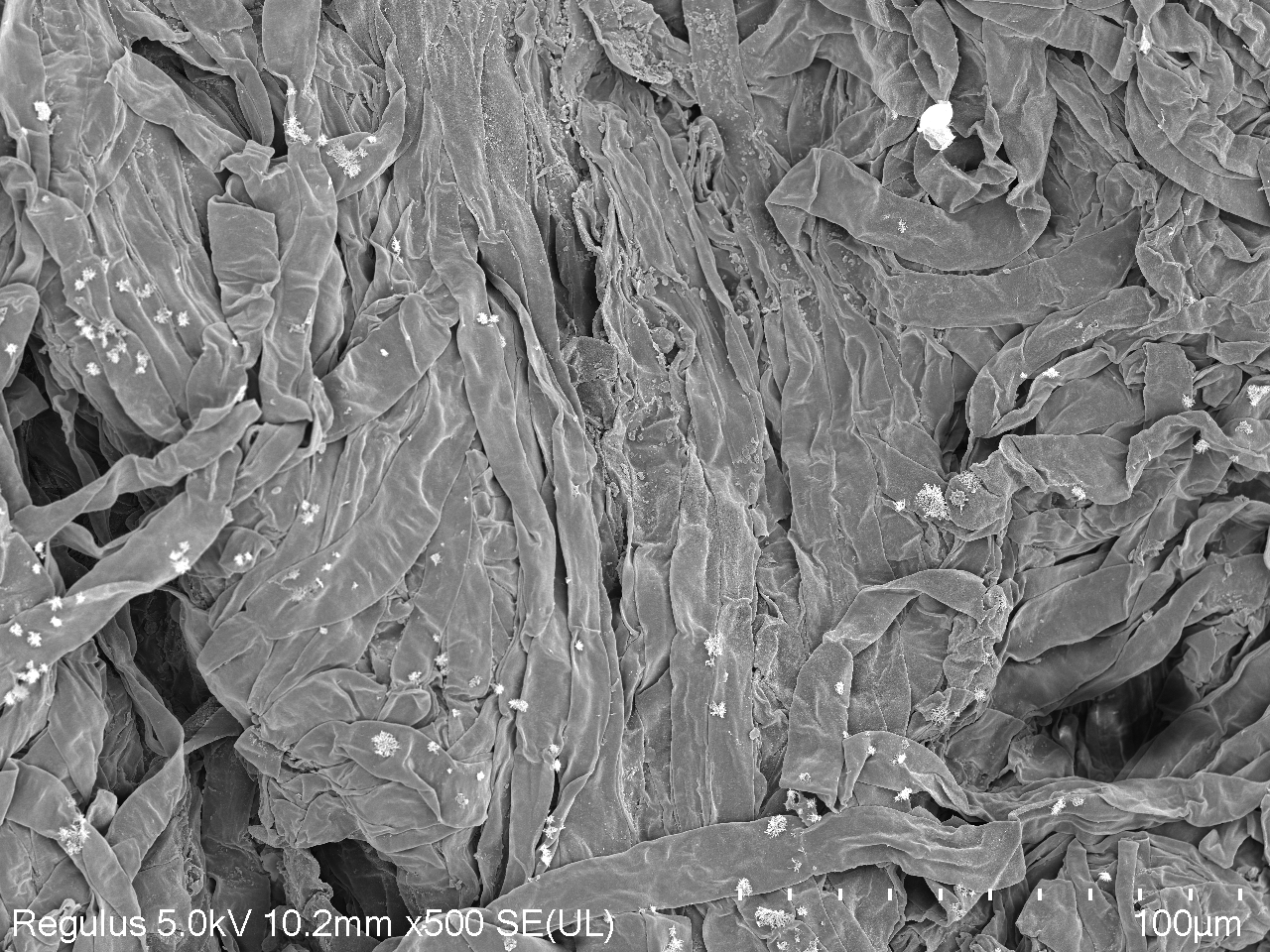

Supplement: Supplementary file 1 [file DataSheet1.zip › SEM/III(day8).tif]
